# Transient Stuttering as a Sole Presentation in a Patient With a Subcentimeter Left Frontal Cortical Ischemic Infarct

**DOI:** 10.7759/cureus.69466

**Published:** 2024-09-15

**Authors:** Ethan Salter, Sophia I Salter, William Im, Aengela J Kim, Rachel Williams, Christina Liou, Stephen Liu, Antonio K Liu

**Affiliations:** 1 Neurology, Adventist Health White Memorial, Los Angeles, USA; 2 Internal Medicine, Adventist Health White Memorial, Los Angeles, USA; 3 Neurology, University of Central Florida, Orlando, USA; 4 Emergency, Adventist Health White Memorial, Los Angeles, USA; 5 Neurology, Loma Linda University School of Medicine, Loma Linda, USA

**Keywords:** subcentimeter, left frontal cortex, acute ischemic stroke (ais), transient, stuttering

## Abstract

Stuttering is a complex speech disorder that is associated with a variety of etiologies, including psychological factors, metabolic disorders, and structural abnormalities. While stuttering is typically not caused by stroke, it is important to include it in the diagnostic evaluation, especially in patients with a history of neurological conditions. Of the clinical presentations of stroke-induced stuttering, transient stuttering following stroke has seldom been documented, as most patients who develop a stutter following a stroke end up developing permanent speech deficits. Additionally, as most cases of stroke-induced stuttering are part of a broader neurological presentation, stuttering generally does not present as an isolated symptom. Furthermore, although strokes of various sizes have been implicated in stuttering, it is quite uncommon for the affected lesion to be smaller than 1 cm. We present a rare case of transient, isolated stuttering following a subcentimeter stroke and a subsequent review of relevant literature. Our case report highlights the diversity of stroke-related speech disorders and the importance of considering even minor strokes in the differential diagnosis of stuttering.

## Introduction

Stuttering is a speech disorder characterized by disruptions in the rate, rhythm, and flow of speech which often leads to speech disfluencies, such as involuntary repetitions of sounds, syllables, or words; prolongations of sounds; or silent or sound-filled blocks where airflow is interrupted. These disruptions can make speaking clearly a challenge, significantly affecting a patient’s ability to communicate. In addition, stuttering can lead to adverse psychological consequences, such as social anxiety, negative self-talk, secondary mannerisms, and avoidance behaviors [1,2]. Therefore, accurately diagnosing and effectively managing stuttering is extremely important for the patient’s overall well-being.

There are two kinds of stuttering, developmental and acquired. Stuttering that develops later in adulthood offers valuable insights into the neural mechanisms underlying fluent speech production. Unlike developmental stuttering, a childhood-onset condition with complex and not fully understood causes, acquired stuttering develops later on in life as a result of either psychological or neurogenic causes [3]. Psychological stuttering is a type of acquired stuttering that results from a variety of psychiatric conditions, such as affective disorders, somatization, and conversion disorder [4]. In contrast, neurogenic stuttering is a type of acquired stuttering that results following an injury to the brain, such as through traumatic brain injury, stroke, degenerative diseases, drug overuse, and HIV/AIDS [4].

Of the causes of neurogenic stuttering, the most common cause is stroke [5]. Strokes occur as a result of a sudden interruption of blood flow to the brain, either a result of a rupture of a blood vessel, known as a hemorrhagic stroke, or by a blockage of a blood vessel, known as an ischemic stroke. In either case, cerebral perfusion is compromised, which can damage the neural networks involved in speech production and fluency, leading to the development of acquired stuttering [5]. Both the location and severity of the stroke damage have been shown to influence the characteristics of this type of stuttering. Among stroke patients, the incidence of stroke-associated stuttering is estimated to be around 5.3%, with a portion (2.5% or more) experiencing persistent stuttering beyond six months [5]. Reports of rapidly self-resolving, transient stuttering following a stroke are far less common.

Importantly, in the vast majority of cases of stuttering following a stroke, stuttering is not an isolated symptom. Rather, stuttering is often accompanied by other co-occurring speech-language disorders, such as aphasia, dysarthria, and apraxia of speech [5]. This makes it challenging to identify whether acquired stuttering following a stroke is a disorder on its own or if it is a result of one of the other speech disorders. Hence, cases of stuttering as an isolated symptom following a stroke provide valuable insight into the etiology of acquired stuttering. In this case report, we present a case of isolated, transient stuttering following a stroke. We hope that by examining the patient's medical history, neurological presentation, and speech characteristics, we can contribute to a deeper understanding of acquired neurogenic stuttering.

## Case presentation

A 52-year-old obese woman with no significant past medical history presented to the Emergency Department with sudden-onset stuttering that began one hour before arrival. She reported no significant past medical history or medication use. There was no recent illness or sick contact. Family and social history were unremarkable. Her vital signs were stable. Examination revealed an obese lady without any distress. She was pleasant, coherent, oriented, and cooperative. Besides speech examination, her cranial nerves 2 to 12 were intact. She had significant difficulty initiating speech. She had to rapidly repeat the first syllable of her words several times before she could articulate the word. Following this initial repetition, she could speak the rest of the word fluently with normal pronunciation, a feature suggestive of stuttering. She had no difficulty with finding words, repeating sentences, or comprehension. She was also able to name 15 out of 15 objects (from common to rare). She had no dysphagia, facial droop, or extraocular movement issues. Her facial sensation was intact and she reported no tinnitus. A motor examination revealed that she had normal muscle tone, bulk, and strength. She had normal sensations throughout her body and showed no dysmetria, dysdiadochokinesia, or gait abnormality. There was no urine nor bowel incontinence or any frontal release signs. Reflexes were normal throughout.

**Figure 1:**
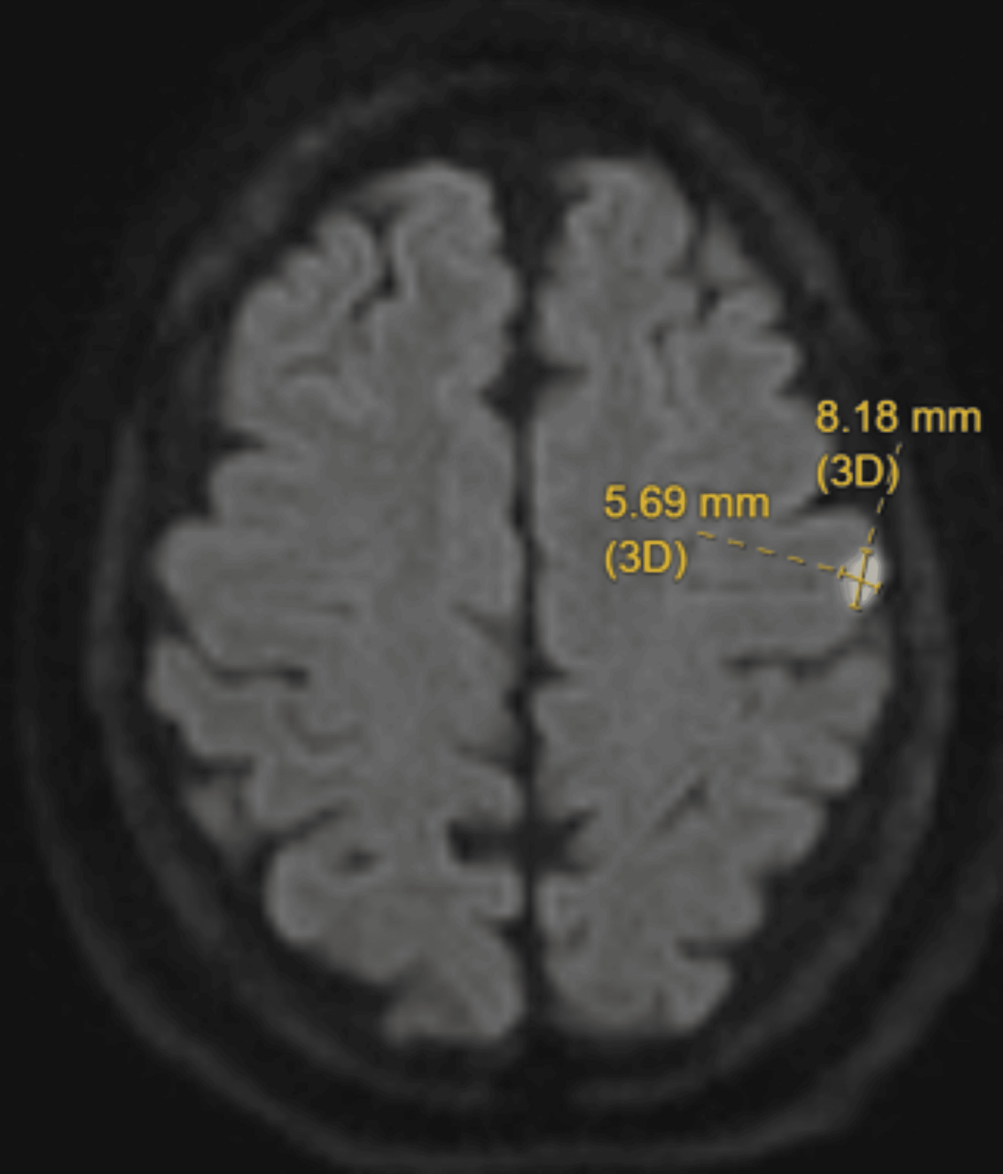
MRI DWI sequence showed a 5.69 x 8.18 mm acute ischemic stroke in the left frontal lobe anterior to the left central sulcus DWI: Diffusion-weighted imaging

**Figure 2:**
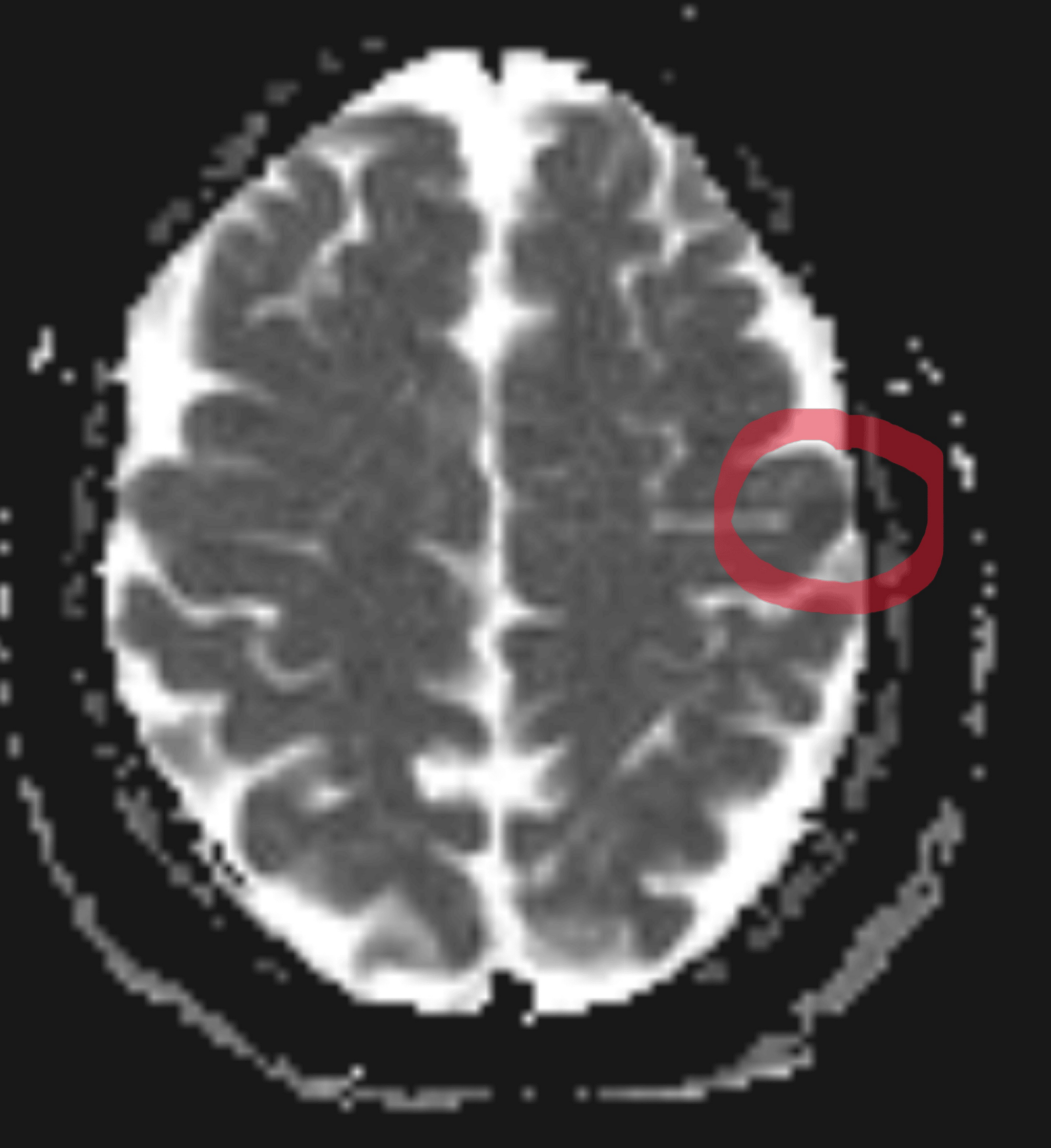
MRI ADC sequence showed an area of hypoperfusion at the same location of the DWI abnormality ADC: Apparent diffusion coefficient; DWI: diffusion-weighted imaging

Importantly, her stuttering resolved within about 2 hours of presentation. Since then, all neurological examinations have been normal. Laboratory investigation yielded no relevant findings. She tested negative for COVID-19. The toxicology study was negative. Imaging studies, including a head CT scan and CT angiography of the head and neck, were unremarkable. However, MRI revealed a small area of abnormal signal on diffusion-weighted imaging (DWI), consistent with a small acute ischemic stroke in the left frontal cortex (Figures 1-2). A stroke workup found no underlying cause. Antithrombin III panel, activated protein C resistance, antiphospholipid antibody panel, protein C profile, and protein S profile were all negative. Cardiac workup including echocardiogram (ejection fraction 60%) and cardiology consult were nonrevealing. The patient was discharged on a daily oral regimen of aspirin (81 mg), Plavix (75 mg), and Lipitor (40 mg) for stroke prevention. Prolonged outpatient cardiac rhythm monitoring over three months yielded no signs of atrial fibrillation.

## Discussion

Despite our patient’s symptoms lasting for only two hours or so, the final diagnosis is a small acute stroke, not a transient ischemic attack (TIA). This is evident by the positive DWI on the brain MRI. However, our patient’s case is unique in three ways: (1) stuttering was an isolated symptom; (2) the stuttering was transient, having been resolved in the time frame typically seen in a TIA; and (3) the stroke itself was only 8 mm in size.

Our literature search, after eliminating cross-reference cases, identified one extensive systematic review [6] and four additional case reports published in recent years on stuttering as a stroke symptom [7-10]. The most extensive study is from Saez-Calveras et al. [6], which reviewed 63 patients from previous studies in addition to their own case report. They did a rather extensive systematic review, listing two prospective studies, 11 case reports, and four case series from 1978 to 2016. All the cases they included will be excluded from being referenced individually unless specifically discussed.

We reviewed all the articles and cases listed in Saez-Calveras’ article plus the four extra case reports we found. Our analysis focused on three key features: (1) whether stuttering was isolated, (2) whether stuttering was transient, and (3) the size and location of any lesions. We found out the following: the two prospective studies in Saez-Calveras were both written by Theys in 2011 and 2013 [5,11]. Their studies listed 17 and 20 stroke subjects with neurogenic stuttering, respectively. However, Theys’ 2013 paper reported that 31 of the 37 total patients (20 stroke subjects with stuttering plus 17 without stuttering) were previously published in the 2011 paper. To avoid double counting these patients, we considered only 20 total stroke subjects with neurogenic stuttering patients from Theys (instead of 17 plus 20). Furthermore, in one of the cases listed in Saez-Calveras’ paper written by Kakishita in 2004 [12], there were five cases instead of one. Taking these factors into consideration, our analysis is based on a total of 51 patients.

In reviewing these cases to determine if symptoms were isolated, only 46 cases (among the 51 cases reported) have detailed enough symptom descriptions for analysis. Of these 46 cases, only the case reported by Saez-Calveras [6] describes an isolated case of stuttering. There are two other potentially isolated presentations among the 17 patients reported by Theys. However, these cases only look at language and speech areas, with no mention of body weakness, leaving their isolated status uncertain. The remaining 43 cases displayed various co-occurring symptoms (counting the most prominent symptom so each case is counted once only), including right body weakness (15), aphasic and/or dysarthria (15), left weakness (six), apraxia (three), dysphagia (one), vertical nystagmus (one), alexia (one), decreased mentation together with restless leg (one). In other words, only a single case is similar to our reported case of being isolated in presentation without any other usual stroke symptoms.

Next, we reviewed cases based on the symptom duration, looking for other cases that resolved quickly. Some of these reports explicitly stated the duration of symptoms, but for many other cases, we can safely estimate from their enrollment criteria that they were much longer than two hours. Of the 51 patients analyzed, only 42 cases have clear reference to the duration of the symptoms. Among these 42 patients, only three were labeled transient. Saez-Calveras reported episodic, recurrent stuttering episodes, each lasting only 10 seconds [6]. Nass reported a two year old with transient stuttering, but the specific duration was not available [13]. Grant reported a transient stuttering that lasted a week [14] (both Nass and Grant’s paper were included in Saez-Calveras paper). Theys’ study only provides duration data for 14 patients. Six of these cases were reported to have been resolved within six months. However, their recruitment method, with speech pathologist enrolling subjects from an inpatient population, implies that all 20 patients, regardless of whether or not the duration was documented, likely experienced symptoms for more than two hours. Of these cases, our patient's stuttering seemed to resolve at a time frame most similar to that of Saez-Calveras’ patient. However, our patient's case aligns more closely with a TIA presentation, whereas Saez-Calveras’ patient had multiple episodic stuttering lasting seconds.

Finally, we reviewed cases based on the location and size of the lesion, despite this data being the hardest to access. There were no MRI reports before 1985, so many cases reported CT findings only. Additionally, many other cases only verbally reported the lesion location without any picture or size data. While They's paper from 2013 offers a lesion overlay diagram [11], it is difficult to determine individual lesion size due to the inclusion of 20 lesions being overlaid in the same diagram. Saez-Calveras' paper [6] presents the only other case with a subcentimeter stroke. However, there is a significant difference in that our case involves a small lesion in the cortical area of the left frontal lobe, whereas their patient’s lesion is located in the left precuneus cortex.

After reviewing all the available literature, only the study by Saez-Calveras et al. presented a case somewhat similar to our patient. The condition in this report was isolated, brief, transient, and subcentimeter in size. However, the location of the patient’s lesion differed from that of our patient, and the presentation was episodic, occurring in three separate 10-second instances, rather than lasting for two hours. Additionally, neither the case reported by Saez-Calveras et al. nor our patient's presentation aligns with the typical locations identified in previous literature, such as the cortical-striatal-cortical loop as reported by Theys in 2013 [11]; the left putamen, claustrum, and amygdalostriatal transition area as reported by Theys in 2024 [15]; and the frontostriatal tract and frontal aslant tract [16,17]. The underlying pathophysiology between a small left frontal cortical stroke and transient stuttering remains elusive. It is consistent with the suggestion and observation by Saez-Calveras et al. that stuttering can be a result of disruption anywhere in a cortico-striato-cortical integrative pathway [6]. 

## Conclusions

This report presents a rare case of a 52-year-old woman who developed isolated, transiently resolving stuttering following a small acute ischemic stroke. The absence of co-occurring speech-language disorders, coupled with symptom duration lasting only two hours, and the subcentimeter size of the lesion sheds insight into the highly variable symptom presentation of acquired stuttering following a stroke.

From a clinical perspective, isolated transient stuttering without any other language deficit that resembles a TIA can be the result of a subcentimeter small stroke. Careful examination is needed to ensure proper diagnosis so that proper stroke management can be quickly initiated.
